# Examining the development of Alzheimer's disease‐like symptoms in a rabbit model of cerebral palsy

**DOI:** 10.1002/alz70855_103327

**Published:** 2025-12-23

**Authors:** Landon Genry, Elvia Mena Avila, Emily J Reedich, Scott Matson, Ashlee Davis, Lisa Dowaliby, Katharina A Quinlan

**Affiliations:** ^1^ University of Rhode Island, Kingston, RI, USA; ^2^ Ryan Institute, Kingston, RI, USA

## Abstract

**Background:**

Cerebral Palsy (CP) is the most common motor disability in children and affects 1.5 ‐ 2.5 out of every 1,000 live births. Adults with CP have poorer general cardiovascular health and higher total cholesterol levels than those without CP. These modifiable lifestyle factors could contribute to an increased risk for Alzheimer's disease (AD). Recent evidence confirms that people with CP are at a higher risk for developing AD and related dementias as they age. However, the nature of this link is still unclear: do early life stressors/environmental insults, like those associated with CP, cause changes in the brain that may lead to the development of disease later in life, or does the presence of modifiable lifestyle factors increase risk?

**Methods:**

To address this question, we combined the high‐cholesterol‐fed rabbit model of AD combined with the prenatal hypoxia‐ischemia (HI) model of CP. At 70‐80% gestation, blood flow was restricted to the uterus of a pregnant dam for 40 minutes causing global hypoxia in the rabbit kits *in utero*. Rabbit kits were born naturally at term. At weaning, rabbits were placed on a normal diet or a high cholesterol diet with copper in the drinking water. After 6 months on the diet, we performed trace eyeblink conditioning, object location memory, and novel object recognition to assess cognitive function. We also measured changes in protein expression to examine histopathological changes consistent with AD.

**Results:**

We found a significant positive correlation between serum cholesterol levels and the length of time it took the rabbits to reach the threshold for learning in trace eyeblink conditioning. In novel object recognition, there is a main effect of HI. HI rabbits have a decreased preference for the object in a new location. When rabbits are fed a high cholesterol diet, HI leads to an increase in APP expression in the frontal cortex, but there are no differences between groups in GFAP expression.

**Conclusion:**

More work needs to be done to clarify the relationship between prenatal brain injury and susceptibility to AD, but our results support a role of early life brain injuries contributing to risk of AD.

